# Prognostic Value of ^18^F-FDG and ^18^F-FEC Uptake in Hepatocellular Carcinoma Using Contrast-Enhanced Integrated PET/MRI: Correlation with Histology and Survival

**DOI:** 10.3390/cancers18101526

**Published:** 2026-05-09

**Authors:** Marzieh Nejabat, Lucian Beer, Theresa Servus, Ahmed Ba-Ssalamah, Peter Mazal, Lukas Nics, Marcus Hacker, Georgios Karanikas, Sazan Rasul

**Affiliations:** 1 Department of Biomedical Imaging and Image-Guided Therapy, Division of Nuclear Medicine, Medical University of Vienna, 1090 Vienna, Austria; marzieh.nejabat@meduniwien.ac.at (M.N.); theresa.servus@gmail.com (T.S.); lukas.nics@meduniwien.ac.at (L.N.); marcus.hacker@meduniwien.ac.at (M.H.); georgios.karanikas@meduniwien.ac.at (G.K.); 2 Department of Biomedical Imaging and Image-Guided Therapy, Division of General and Pediatric Radiology, Medical University of Vienna, 1090 Vienna, Austria; lucian.beer@meduniwien.ac.at (L.B.); ahmed.ba-ssalamah@meduniwien.ac.at (A.B.-S.); 3 Department of Pathology, Medical University of Vienna, 1090 Vienna, Austria; peter.mazal@meduniwien.ac.at

**Keywords:** dual-tracer PET imaging, alpha-fetoprotein, molecular imaging, tumor biology, tumor prognosis

## Abstract

**Background:** Dual-tracer positron emission tomography/magnetic resonance imaging (PET/MRI) using [^18^F]-fluorodeoxyglucose (^18^F-FDG) and [^18^F]-fluoroethylcholine (^18^F-FEC) may reveal complementary aspects of hepatocellular carcinoma (HCC) biology. This retrospective study evaluates whether PET parameters obtained from ^18^F-FDG and ^18^F-FEC correlate with MRI enhancement parameters, tumor histological grade, and survival in patients with HCC. **Methods:** We retrospectively evaluated 25 patients who underwent integrated PET/MRI. Lesions were analyzed on pre-contrast MRI and at early (3–5 min) and late (20–30 min) post-contrast phases. For ^18^F-FDG and ^18^F-FEC, standardized uptake values (SUV maximum, mean, and peak) and metabolic tumor volume were measured, along with lesion size, MRI enhancement ratios, AFP levels, and survival. For patient-level imaging analyses, the largest lesion per patient was defined as the index lesion. Correlation analysis using Spearman’s rank correlation coefficient, Kaplan–Meier analysis, log-rank testing, and exploratory Cox proportional hazards models were performed. **Results:** ^18^F-FDG SUVmean (index lesion) showed the strongest inverse association with survival (r = −0.61, *p* = 0.003), followed by SUVpeak (r = −0.50, *p* = 0.012). ^18^F-FDG SUVmean differed across tumor grades (G1–G3; *p* = 0.040), without a consistent trend. In Cox regression, ^18^F-FDG SUVpeak was significantly associated with shorter overall survival (HR 1.22, 95% CI 1.05–1.42, *p* = 0.01), whereas SUVmean showed only a borderline association (*p* = 0.07). When split at the median, SUVmean was also significantly associated with shorter survival (*p* < 0.001; HR 10.39, 95% CI 2.75–39.3). ^18^F-FEC parameters were not associated with survival but showed a moderate correlation with AFP levels (r = 0.41, *p* = 0.01). Dynamic MRI early and delayed enhancement of the index lesion were strongly correlated (r = 0.70, *p* < 0.001) but not associated with survival. **Conclusions:** In HCC, ^18^F-FDG uptake provides prognostic information beyond MRI enhancement and histology, reflecting tumor aggressiveness and independently predicting survival. While ^18^F-FEC-PET complements lesion characterization and correlates with AFP, it does not show meaningful prognostic value. MRI enhancement parameters were not associated with survival in this cohort. ^18^F-FDG-based metabolic imaging may improve pre-treatment risk stratification, whereas dual-tracer PET/MRI should be considered a selective, exploratory approach rather than routine imaging.

## Simple Summary

Patients with hepatocellular carcinoma (HCC) have a poor prognosis, partly due to their biological heterogeneity, as morphology and vascularity do not always reflect metabolic aggressiveness or the histological grade of the disease. Here, we conducted a retrospective, patient-level analysis of 47 HCC lesions in 25 patients who underwent contrast-enhanced PET/MRI with dual tracers (^18^F-FDG and ^18^F-FEC). In these patients, ^18^F-FDG uptake provided prognostic information beyond MRI and histology, reflecting tumor aggressiveness and independently predicting patient survival. In contrast, ^18^F-FEC PET supported lesion characterization and correlated with alpha-fetoprotein levels but showed no significant prognostic value. The ^18^F-FDG signal appeared higher in pre-treatment patients, indicating that treatment status may influence the interpretation of results. These findings suggest that ^18^F-FDG PET/MRI is well suited for staging and prognostication in newly diagnosed HCC, while dual-tracer imaging with ^18^F-FDG and ^18^F-FEC may be reserved for selected high-risk or diagnostically challenging cases. Further prospective studies with larger sample sizes are needed to define the role of ^18^F-FDG PET/MRI in the clinical management of patients with HCC.

## 1. Introduction

Hepatocellular carcinoma (HCC) remains the third leading cause of cancer-related mortality worldwide due to its increasing incidence, particularly in regions with a high prevalence of metabolic dysfunction-associated steatotic liver disease, alcohol-related liver disease, and chronic viral hepatitis [1]. Despite advances in surveillance and therapy, the prognosis for many patients is still poor, largely because HCC is a biologically heterogeneous tumor and its morphology and vascularity do not always reflect its metabolic aggressiveness or histological grade.

Conventional imaging modalities, particularly contrast-enhanced computed tomography (CT) and magnetic resonance imaging (MRI), remain the cornerstone for hepatocellular carcinoma (HCC) detection. These techniques enable visualization of characteristic morphological and perfusion patterns, including arterial phase hyperenhancement and subsequent venous washout, permitting non-invasive diagnosis in accordance with LI-RADS criteria [2,3]. Nonetheless, such imaging features provide only a limited representation of intra-tumoral heterogeneity and are insufficient for accurate prognostication. For example, poorly differentiated HCCs may exhibit diminished arterial enhancement despite heightened metabolic activity, thereby reducing the predictive utility of CT and MRI findings alone.

Alternative approaches, such as the Barcelona Clinic Liver Cancer (BCLC) staging system, are commonly used to estimate prognosis and guide therapeutic decision-making [4]; however, their utility is limited to patients who have undergone initial staging. Positron emission tomography (PET) provides functional information that complements the structural detail of conventional imaging modalities [5,6]. Recent investigations have aimed to clarify the role of PET not only in the detection and prognostic evaluation of HCC lesions but also in the advancement of personalized treatment strategies. In this context, current evidence highlights that metabolic imaging in HCC should primarily serve to guide treatment selection, monitor therapeutic response, and predict clinical outcomes, rather than focusing solely on lesion detection sensitivity [7,8,9,10,11].

Indeed, PET tracers such as [^18^F]-fluorodeoxyglucose (^18^F-FDG) and [^18^F]-fluoroethylcholine (^18^F-FEC) visualize and characterize distinct metabolic processes. Specifically, ^18^F-FDG measures glycolytic activity and has been shown to be prognostically valuable, whereas ^18^F-FEC, a choline analog, reflects cell membrane synthesis and can detect well-differentiated lesions. When combined with MRI, dual-tracer PET may characterize both metabolic and proliferative aspects of HCC. Most MRI protocols rely on dynamic imaging with late-arterial, portal-venous and delayed phases [12,13,14,15,16,17].

In this study, we sought to investigate the relationship between ^18^F-FDG and ^18^F-FEC uptake and various dynamic and non-dynamic MRI parameters, as well as to assess whether these imaging biomarkers provide complementary prognostic information. To this end, patients with HCC who underwent integrated PET/MRI with both ^18^F-FDG and ^18^F-FEC were analyzed. The objective was to determine the degree of correlation between the two tracers, their association with MRI features (specifically T1 VIBE enhancement at 2 and 20–30 min), and their relationship with indicators of biological tumor aggressiveness, including histological grade, alpha-fetoprotein (AFP) levels, and overall survival (OS).

## 2. Materials and Methods

### 2.1. Patients and Study Design

We conducted a retrospective analysis of twenty-five patients at various stages of the disease who underwent contrast-enhanced integrated PET/MRI. Patients included in the study had confirmed pathology reports of HCC and were excluded if they had any secondary malignancies. Furthermore, patients were excluded if clinical and laboratory data were unavailable or if the quality of the imaging was deemed unacceptable. All imaging was performed according to a standardized protocol to ensure consistency. Pathology reports were classified based on histological grade as well-differentiated (G1), moderately differentiated (G2), or poorly differentiated (G3). Comprehensive laboratory, histological, and survival data were collected for all participants. Informed consent for imaging was obtained from all patients, and the study received approval from the institutional review board.

### 2.2. PET and MRI Imaging Protocol

All examinations were conducted on an integrated whole-body PET/MRI scanner equipped with a 3-Tesla magnetic field (Biograph mMR; Siemens Healthcare, Germany). For the PET component, ^18^F-FDG was administered intravenously at a dose of 3–5 MBq/kg of body weight. Following an uptake period of 45–60 min, a whole-body PET/MRI scan was acquired in accordance with the institution’s standard liver MRI protocol. Upon completion of the initial imaging, ^18^F-FEC was administered at a dose of 3–4 MBq/kg of body weight, followed by a dedicated dynamic PET liver acquisition.

The MRI protocol commenced with the acquisition of standard anatomical sequences. Subsequently, 10 mL of gadoxetic acid (Primovist^®^; Bayer Healthcare, Germany), a gadolinium-based hepatobiliary contrast agent, was administered intravenously. T1-weighted volumetric interpolated breath-hold examination (VIBE) sequences were obtained at three predefined time points: pre-contrast (native), early post-contrast (3–5 min), and delayed post-contrast (20–30 min).

### 2.3. PET- and MRI-Image Analysis

PET images were analyzed using Hermes Hybrid 3D software (version 4.17, Hermes Medical Solutions, Stockholm, Sweden) [18]. The software automatically calculated standardized uptake values (SUVs). For each tracer and lesion, maximum, mean, and peak standardized uptake values (SUVmax, SUVmean, and SUVpeak) were extracted. Total metabolic tumor volume (MTV) was calculated by summing all metabolically active lesions per patient.

In addition, visual and quantitative analyses of MRI images were performed using IMPAX Volume Viewing software (version 3.0, AGFA Healthcare, Germany), which enables side-by-side visualization and fusion of PET and MRI datasets. Hepatic tumor diameters were measured on Pet/MRI images using a metabolic axial approximation, supported by MRI soft-tissue alterations of the corresponding ^18^F-FDG- or ^18^F-FEC-positive lesions, where applicable. Lesions suspicious for HCC on PET were defined as intrahepatic lesions positive for either tracer, demonstrating visually higher ^18^F-FDG or ^18^F-FEC uptake than the surrounding hepatic background. For the FDG-positive lesion with the highest uptake, signal intensity was measured on T1-weighted VIBE sequences at pre-contrast, early post-contrast (3–5 min), and late post-contrast (20–30 min) time points.

### 2.4. Variables and Statistical Analysis

Primary variables included lesion diameter, MRI enhancement values at the aforementioned time points, SUVmax, SUVmean, and SUVpeak of ^18^F-FDG and ^18^F-FEC, histological grade, survival, and AFP concentration. Normally distributed variables were presented as mean ± standard deviation (SD); otherwise, as median and range. To maintain statistical independence, all analyses were performed at the patient level (*n* = 25). In patients with multifocal disease, the largest hepatic lesion (index lesion) was selected as representative. Spearman’s rank correlation coefficient (r) was used to assess associations between imaging parameters and clinical or laboratory variables, with results visualized as a heatmap. Differences in SUV across histological grades were explored using box plots and compared with the Kruskal–Wallis test.

Survival time was defined from the date of PET/MRI examination to death or last follow-up. Moreover, survival was analyzed using Kaplan–Meier curves, with patients categorized into high- and low-uptake groups based on the median SUV of the index lesion. Cox proportional hazards regression was used for exploratory univariable analyses. Due to the collinearity between SUV metrics and the small sample size, only restricted multivariable models were constructed. Proportional hazards assumptions were verified by inspecting Schoenfeld residual patterns, which showed no significant violations. No formal adjustment for multiple comparisons was applied; therefore, all findings should be considered exploratory. Statistical analyses were performed using Python (version 3.14) and R (version 4.6), with significance set at *p* < 0.05.

## 3. Results

### 3.1. Patient and Tumor Characteristics

As shown in Table 1, the dataset comprised 25 patients (18 men and seven women): 33 G1 lesions, four G2 lesions, and 10 G3 lesions. The median lesion diameter was 3.3 cm (range 0.9–17.7 cm) and the median survival was 1034 days (range 23–3551 days). The median ^18^F-FDG SUVmax was 5 (range 3.5–8.2), and the median ^18^F-FEC SUVmax was 12.5. Furthermore, the median MTV for ^18^F-FDG was 80.4 cm^3^ and for ^18^F-FEC it was 90.7 cm^3^. AFP levels varied widely with a mean ± SD of 189 ± 436 and a median of 7.8 IU/mL.

### 3.2. Correlation Analysis

**Figure 1:**
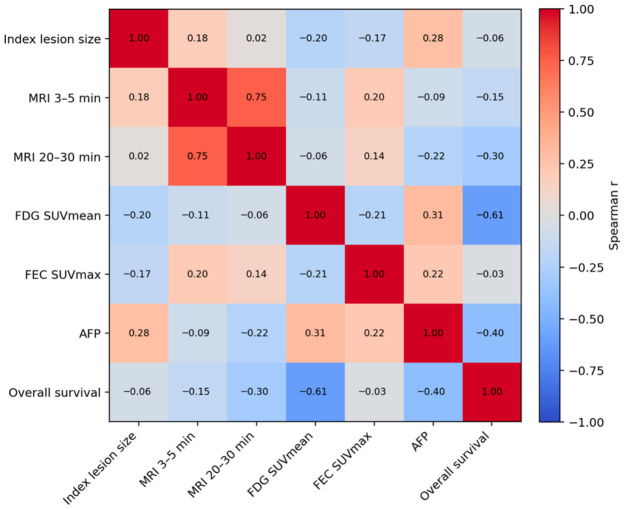
Spearman correlation heatmap of lesion diameter, MRI enhancement (native, 3–5 min, 20–30 min), ^18^F-FDG SUVmax, ^18^F-FEC SUVmax, survival and AFP. Warmer colors indicate positive correlations and cooler colors indicate negative correlations. The strongest adverse association with overall survival was observed for ^18^F-FDG SUVmean, whereas early and delayed MRI enhancement were strongly interrelated.

^18^F-FDG SUVmean showed the strongest inverse association with overall survival (r = −0.61, *p* = 0.003), followed by SUVpeak (r = −0.50, *p* = 0.012). ^18^F-FDG SUVmax showed only a borderline inverse association with survival (r = −0.38, *p* = 0.08). In contrast, ^18^F-FEC SUVmax was not associated with survival (r = −0.03, *p* = 0.93), nor were the ^18^F-FDG or ^18^F-FEC MTV values. Moreover, ^18^F-FEC SUVmax showed a positive correlation with AFP levels (r = 0.22, *p* = 0.01). Spearman correlations among the most relevant numeric variables are illustrated in Figure 1.

The MTV of ^18^F-FDG showed a weak but statistically significant negative correlation with survival (r = −0.35, *p* < 0.05). Furthermore, ^18^F-FDG uptake correlated moderately with ^18^F-FEC (r = 0.21) and AFP (r = 0.31).

Importantly, neither the ^18^F-FDG nor ^18^F-FEC SUV values correlated significantly with MRI enhancement parameters (native, 3–5 min, or 20–30 min; all *r* < 0.20, *p* > 0.05). However, the MTV of ^18^F-FDG and ^18^F-FEC showed weak correlation with contrast enhancement at 3 min (*r* = 0.35, *p* = 0.02 and r = 0.42, *p* = 0.01, respectively). The MTV of ^18^F-FDG and ^18^F-FEC correlated strongly with each other (r = 0.85, *p* = 0.0001), but neither correlated significantly with the late MRI contrast enhancement parameters.

Furthermore, lesion size showed no association with either ^18^F-FDG or ^18^F-FEC uptake (SUVmax, SUVmean, SUVpeak; all *p* > 0.05), indicating that metabolic activity was independent of tumor size in this cohort.

### 3.3. Image Parametric and Tumor Histological Grade

**Figure 2:**
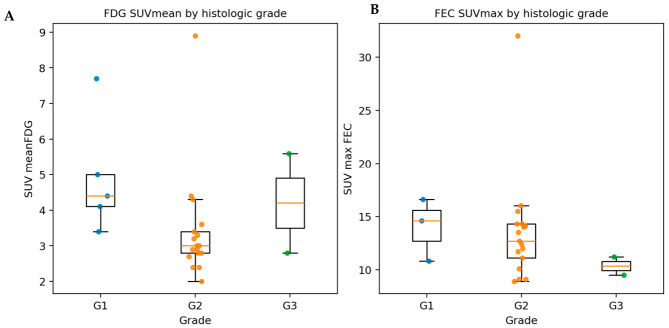
SUVmean values of ^18^F-FDG and ^18^F-FEC and histological grading of the tumor. (**A**) ^18^F-FDG SUVmean is significantly higher in poorly differentiated (G3) lesions than in well-differentiated (G1–G2) lesions. (**B**) ^18^F-FEC SUVmax demonstrates limited grade-dependent variation, with considerable overlap between G1–G2 and G3 lesions.

As shown in Figure 2, ^18^F-FDG SUVmean showed a significant trend across the recorded grades G1–G3 (Kruskal–Wallis *p* = 0.04). The ^18^F-FEC SUV values, including SUVmax, did not differ significantly across grades (all *p* > 0.05). Moreover, MRI enhancement parameters (native, 3–5 min, and 20–30 min) showed no significant correlation with tumor grade (all *p* > 0.05).

### 3.4. Survival Analysis

In univariable Cox analysis, ^18^F-FDG SUVpeak was the strongest PET predictor of shorter overall survival (HR 1.22, 95% CI 1.05–1.42, *p* = 0.01). SUVmean showed a borderline association (HR 1.22, 95% CI 0.98–1.53, *p* = 0.07), whereas ^18^F-FDG SUVmax, all ^18^F-FEC SUV parameters, MRI measurements, and index lesion diameter were not significant. Treatment status also showed a borderline adverse association with survival, with shorter survival after post-treatment imaging (HR 2.71, 95% CI 0.97–7.54, *p* = 0.06), as shown in Table 2.

**Figure 3:**
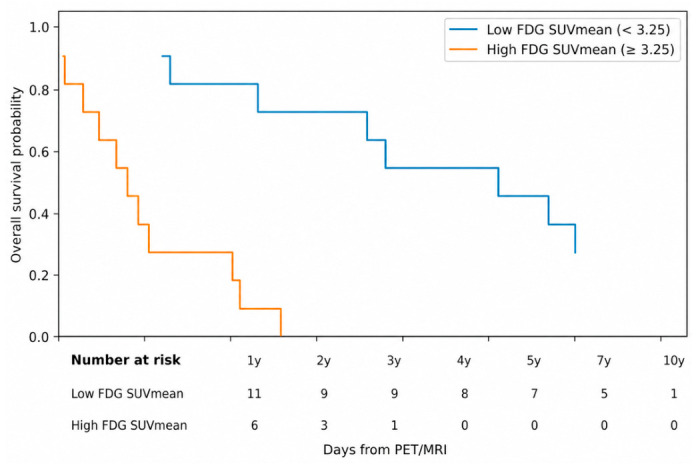
Kaplan–Meier survival curve for ^18^F-FDG SUVmean dichotomized at the median. Numbers at risk, the log-rank *p* value, and the dichotomized hazard ratio with 95% confidence interval are shown. ^18^F-FDG SUVmean demonstrated clear patient-level survival separation.

In Kaplan–Meier analysis, patients with high ^18^F-FDG SUVmean (≥3.25) had significantly shorter survival (log-rank *p* < 0.001; HR 10.39, 95% CI 2.75–39.30; Figure 3). By contrast, ^18^F-FEC variables showed no meaningful survival separation in either correlation or Cox analyses.

In a sensitivity analysis restricted to pre-treatment patients, the prognostic association of ^18^F-FDG became more pronounced. Among 11 pre-treatment patients with complete survival time data, ^18^F-FDG SUVmean showed a strong inverse correlation with overall survival (Spearman r = −0.75, *p* = 0.008). ^18^F-FDG SUVpeak showed a similar trend (r = −0.57, *p* = 0.065), whereas SUVmax and MTV were not significantly correlated with survival. Kaplan–Meier analysis using median dichotomization demonstrated significantly shorter survival in patients with high pre-treatment ^18^F-FDG SUVmean (log-rank *p* = 0.04). In contrast, none of the ^18^F-FEC parameters showed a significant association with survival in the pre-treatment subgroup (all *p* > 0.10).

## 4. Discussion

This study shows that ^18^F-FDG uptake is associated with shorter survival in patients with histologically confirmed HCC, whereas ^18^F-FEC is not. The association was most consistent for SUVmean and SUVpeak. ^18^F-FDG SUVmean demonstrated the clearest monotonic association with survival and the strongest separation in Kaplan–Meier analysis, while SUVpeak showed the strongest association in continuous Cox regression. These findings are biologically plausible and consistent with prior reports linking ^18^F-FDG avidity to aggressive HCC behavior and poor outcomes [8,9,10,11]. However, ^18^F-FEC did not demonstrate prognostic value after patient-level reanalysis, in line with previous studies suggesting that choline-based tracers are useful for lesion characterization and complementary detection but provide limited prognostic discrimination [13,14,15].

In our cohort, ^18^F-FDG SUVmean differed across tumor grades, with values approximately doubling from intermediate-grade (G2) to high-grade (G3) HCC and was associated with shorter survival. These findings are consistent with prior reports indicating that ^18^F-FDG-avid HCCs exhibit aggressive biological behavior. For example, Ida et al. reported a 5-year survival rate of 22% in ^18^F-FDG-positive small HCCs compared with 88% in ^18^F-FDG-negative lesions [8]. ^18^F-FDG positivity has also been associated with microvascular invasion and early postoperative recurrence. In this context, Wang et al. demonstrated that increased ^18^F-FDG metabolic activity (SUV ratio > 2), together with elevated AFP, independently predicted microvascular invasion. Taken together, these findings support the role of ^18^F-FDG PET as a noninvasive biomarker of tumor aggressiveness and prognosis in HCC. From a clinical perspective, ^18^F-FDG-avid HCC should be considered a subtype that warrants closer surveillance or aggressive therapy. In contrast, ^18^F-FDG-negative tumors, which are often well-differentiated, tend to follow a more indolent course [10].

By contrast, ^18^F-FEC uptake demonstrated only a modest association with serum AFP levels and showed no independent relationship with survival. In our cohort, ^18^F-FEC SUV correlated moderately with AFP (r = 0.44) but exhibited no meaningful prognostic association (r = −0.05) with survival. These findings suggest that increased choline metabolism reflects biologically active tumor tissue, often accompanied by elevated AFP, but does not necessarily correspond to an aggressive or lethal tumor phenotype. Consistent with prior reports, ^18^F-FEC PET has been shown to be highly sensitive for the detection of well-differentiated HCC, which may be occult on ^18^F-FDG imaging [11]. For instance, Talbot et al. and others demonstrated that ^18^F-fluorocholine detects the majority of well-differentiated HCC lesions, whereas ^18^F-FDG frequently fails to identify these tumors [12].

On the other hand, the sensitivity analysis in pre-treatment patients suggests that the prognostic value of ^18^F-FDG is stronger before therapeutic intervention, when tracer uptake is less likely to be confounded by treatment-related changes in tumor biology and morphology [14]. In this subgroup, ^18^F-FDG SUVmean showed the strongest association with overall survival [9], whereas ^18^F-FEC remained non-significant. These findings support the interpretation that ^18^F-FDG PET may be most informative for baseline risk stratification in newly diagnosed HCC [9,11], while mixed pre- and post-treatment cohorts may attenuate this signal.

Consistent with this paradigm, Ghidaglia et al. reported that more than half of well-differentiated HCCs were ^18^F-FEC-positive and ^18^F-FDG-negative, while poorly differentiated tumors were more frequently ^18^F-FDG-avid [15]. Accordingly, dual-tracer PET captures a broader metabolic spectrum than either tracer alone, a finding supported by our observation that each tracer detected lesions missed by the other [19,20,21,22,23].

Indeed, neither ^18^F-FDG nor ^18^F-FEC uptake correlated with MRI dynamic enhancement parameters in our study. Although pre-contrast and delayed T1 enhancement ratios were strongly interrelated, they showed no association with PET metrics, indicating metabolic–perfusion uncoupling. This dissociation has been described in HCC, where well-differentiated tumors may remain hypervascular despite low glycolytic activity, whereas dedifferentiated tumors may demonstrate high ^18^F-FDG uptake despite reduced arterial enhancement. These findings indicate that PET and MRI provide largely independent information on tumor biology, supporting the added value of combined PET/MRI for comprehensive disease assessment [24,25,26].

Although tumor size is an established prognostic factor in treatment-naïve HCC [17], this relationship may be attenuated in our mixed cohort, where therapy-induced morphological changes and metabolic heterogeneity obscure size-dependent prognostic associations. Furthermore, the per-patient analytical approach, while allowing assessment of the impact of tumor heterogeneity on survival, may explain why traditional size-based prognostic associations were not observed in our cohort [27,28].

Moreover, evidence from multicenter studies further supports the clinical utility of dual-tracer imaging. For example, Chiu et al. demonstrated that dual-tracer PET/CT with ^18^F-FDG and ^11^C-acetate upstaged approximately 12% of patients and altered management in 8%, with high accuracy for characterizing indeterminate lesions and detecting occult disease in patients with elevated AFP [29]. Similar benefits have been reported in post-therapy settings, where metabolic imaging improves detection of viable tumor after locoregional treatments, despite equivocal morphological findings [25]. Representative prior PET/CT and PET/MRI studies relevant to the results of our present study are summarized in Table 3.

Taken together, these data indicate that ^18^F-FDG PET provides the strongest prognostic information in HCC, whereas ^18^F-FEC primarily enhances lesion detectability, particularly in well-differentiated or post-treatment tumors. While routine dual-tracer imaging may not be cost-effective for all patients, targeted use in high-risk or diagnostically challenging scenarios, such as indeterminate lesions, unexplained AFP elevation, or post-therapy assessment, appears clinically justified [26].

### Limitations

To our knowledge, this is the first study evaluating dual-tracer ^18^F-FEC and ^18^F-FDG PET/MRI in HCC; however, several limitations should be acknowledged. First, it was retrospective, single-center, and based on a small sample size. Second, the cohort included both pre-treatment and post-treatment examinations, although this was addressed in sensitivity analyses. Third, the patient-level analysis was based on the selection of the largest lesion as the index lesion, which avoids non-independence in survival modeling but may underrepresent intra-patient heterogeneity. Fourth, histologic grade was analyzed as recorded and should not be interpreted as lesion-specific pathologic confirmation. Finally, the pre-treatment subgroup analysis was exploratory and based on a small number of patients. Therefore, these findings should be interpreted with caution and require validation in larger prospective cohorts. Future studies incorporating complete clinical staging, longitudinal imaging, advanced quantitative imaging, and radiomic analyses are needed to better define the prognostic value of PET/MRI parameters and their role in monitoring treatment, including dynamic changes in tumor metabolism in HCC.

## 5. Conclusions

Accordingly, ^18^F-FDG PET/MRI appears useful for staging and prognostication in patients with HCC, whereas dual-tracer PET/MRI may be selectively applied in diagnostically challenging or high-risk clinical scenarios where additional metabolic information is likely to influence management. ^18^F-FDG retained a clinically relevant adverse association with survival in this HCC cohort, most clearly for SUVmean in rank-based and Kaplan–Meier analyses and for SUVpeak in continuous Cox modeling. ^18^F-FEC did not show prognostic value. These findings support cautious interpretation and suggest that ^18^F-FDG-based metabolic imaging may add value for pre-treatment risk stratification, whereas dual-tracer PET/MRI should be considered a selective and exploratory approach rather than routine.

### Global Applicability

Where integrated imaging is available, these findings support a cautious, selective use of metabolic imaging in HCC. ^18^F-FDG PET may provide additional biologic risk information beyond morphology, particularly in pre-treatment patients, where MRI-based assessments have limitations. ^18^F-FDG SUV parameters, especially SUVmean, were associated with poorer outcomes and may help to identify higher-risk patients, potentially guiding closer surveillance or more intensive management, independent of tumor size. While ^18^F-FDG appears more relevant for prognostic assessment, ^18^F-FEC may remain useful for lesion characterization in selected cases. However, these data do not support routine dual-tracer PET/MRI for all patients. Its use in clinical practice will depend on availability, logistics, expertise, and cost.

## Figures and Tables

**Table 1:** Clinical and laboratory characteristics of study patients.

Parameters	Values
Patients, *n*	25
Age in years, mean ± SD (range)	66.1 ± 8.8 (52–77)
Sex, *n* (%)	male 18 (72), female 7 (28)
BMI (kg/m^2^), mean ± SD	25.4 ± 3.6
Liver function and laboratory parameters	
AFP (IU/mL), mean ± SD	189 ± 436
ALT (U/L), mean ± SD	61.2 ± 31.7
AST (U/L, mean ± SD	91 ± 98.7
ALP (U/L), mean ± SD	136 ± 67.7
GGT (U/L), mean ± SD	176 ± 141
TB (mg/dL), mean ± SD	0.82 ± 0.5
Albumin (g/L), mean ± SD	40.5 ± 5.6
Clinical status	
* Child–Pugh A, *n* (%)	19 (76)
* Child–Pugh B, *n* (%)	6 (24)
Pre-treatment imaging, *n* (%)	11 (44)
Post-therapeutic imaging, *n* (%)	14 (56)
Alive at last follow-up, *n* (%)	6 (24)
Dead at last follow-up, *n* (%)	19 (76)
Tumor characteristics	
Diameter in cm median	5.8 (2.0–20.0)
G1, *n* (%)	33 (70)
G2, *n* (%)	4 (8.7)
G3, *n* (%)	10 (21.3)

*n*: number; SD: standard deviation; (%): percent; BMI: body mass index; AFP: alpha-fetoprotein; ALT: alanine aminotransferase; AST: aspartate aminotransferase; ALP: alkaline phosphatase; GGT: gamma-glutamyl transferase; TB: total bilirubin; (*): based on patients documented history. G: tumor grading according to WHO classification.

**Table 2:** Exploratory Cox proportional hazards analysis for overall survival.

Variable	HR	95% CI	*p* Value
FDG SUVmean	1.22	0.98–1.53	0.07
FDG SUVpeak	1.22	1.05–1.42	0.01
FDG SUVmax	1.03	0.96–1.11	0.37
FDG MTV	1.00	1.00–1.00	0.23
FEC SUVmax	1.07	0.93–1.22	0.36
FEC SUVmean	1.14	0.98–1.33	0.084
FEC SUVpeak	1.08	0.90–1.29	0.425
FEC MTV	1.00	1.00–1.00	0.826
Histologic grade	0.26	0.05–1.21	0.086
Post-treatment status	2.71	0.97–7.54	0.057
Index lesion diameter	1.08	0.95–1.23	0.243
MRI 20–30 min enhancement	1.01	1.00–1.01	0.226
MRI 3–5 min enhancement	1.00	1.00–1.00	0.962

HR: haz ard ratio; SUV: standardized uptake value; max: maximum; MTV: metabolic tumor volume.

**Table 3:** Selected prior PET/CT and PET/MRI studies relevant to our present study.

Study	Modality	Cohort	Main Finding	Clinical Relevance
Current study	^18^F-FDG and ^18^F-FEC PET/MRI	HCC (pre- and post-treatment)	^18^F-FDG PET useful for staging and prognosis	Supports selective dual-tracer use
Ida et al. [8]	^18^F-FDG PET/CT	HCC after RFA	^18^F-FDG uptake associated with poorer survival	Confirms adverse prognostic role of ^18^F-FDG
Na et al. [9]	^18^F-FDG PET/CT	Advanced HCC	Higher ^18^F-FDG uptake associated with shorter survival	Supports ^18^F-FDG-based survival stratification
Talbot et al. [13]	^18^F-FEC vs. ^18^F-FDG PET/CT	Cirrhosis/CLD	^18^F-FEC improved detection of selected HCC lesions	Complementary detection role of ^18^F-FEC, limited prognostic value
Chiu et al. [29]	^18^F-FDG and ^11^C-acetate PET/CT	HCC staging	Dual-tracer imaging altered clinical management	Supports selective dual-tracer approach
Zucchetta et al. [30]	^18^F-FDG PET/MRI	Post-transplant HCC	Integrated PET/MRI adds value in complex cases	^18^F-FDG PET useful in selected scenarios and surveillance

HCC: hepatocellular carcinoma; RFA: radiofrequency ablation; CLD: chronic liver disease.

## Data Availability

The datasets analyzed during the current study are available from the corresponding author on reasonable request.
